# Integrated and simplified approaches to community management of acute malnutrition in rural Kenya: a cluster randomized trial protocol

**DOI:** 10.1186/s12889-019-7497-3

**Published:** 2019-09-11

**Authors:** Elizabeth Wambui Kimani-Murage, Hermann Pythagore, Elizabeth Mwaniki, Tewoldeberha Daniel, Betty Samburu, Pilar Charle Cuellar, Regina Mbochi, James Njiru, Lucy Wangare, Lydia Karimurio, Olivia Agutu, Lucy Gathigi Maina, Peter Okoth, Judith Raburu, Milka Wanjohi, Triza Macharia, Taddese Alemu Zerfu

**Affiliations:** 1 African Population and Health Research Center (APHRC), Nairobi, Kenya; 2 United Nations Childrens’ Fund (UNICEF), Nairobi, Kenya; 3 Ministry of Health (MOH), Nairobi, Kenya; 4 Action against Hunger (AAH), Madrid, Spain; 5 Save the Children, Nairobi, Kenya; 6 Action against Hunger (AAH), Nairobi, Kenya

**Keywords:** Simplified, Community, Acute malnutrition, Kenya, Trial

## Abstract

**Background:**

In many low income countries, the majority of acutely malnourished children are either brought to the health facility late or never at all due to reasons related to distance and associated costs. Integrated community case management (iCCM) is an integrated approach addressing disease and malnutrition through use of community health volunteers (CHVs) in children under-5 years. Evidence on the potential impact and practical experiences on integrating community-based management of acute malnutrition as part of an iCCM package is not well documented. In this study, we aim to investigate the effectiveness and cost effectiveness of integrating management of acute malnutrition into iCCM.

**Methods:**

This is a two arm parallel groups, non-inferiority cluster randomized community trial (CRT) employing mixed methods approach (both qualitative and quantitative approaches). Baseline and end line data will be collected from eligible (malnourished) mother/caregiver-child dyads. Ten community units (CUs) with a cluster size of 24 study subjects will be randomized to either an intervention (5 CUs) and a control arm (5 CUs). CHV in the control arm, will only screening and refer MAM/SAM cases to the nearby health facility for treatment by healthcare professionals. In the intervention arm, however; CHVs will be trained both to screen/diagnose and also treat moderate acute malnutrition (MAM) and severe acute malnutrition (SAM) without complication. A paired-matching design where each control group will be matched with intervention group with similar characteristics will be matched to ensure balance between the two groups with respect to baseline characteristics. Qualitative data will be collected using key informant and in-depth interviews (KIIs) and focused group discussions (FGDs) to capture the views and experiences of stakeholders.

**Discussion:**

Our proposed intervention is based on an innovative approach of integrating and simplifying SAM and MAM management through CHWs bring the services closer to the community. The trial has received ethical approval from the Ethics Committee of AMREF Health Africa - Ethical and Scientific Review Committee (AMREF- ESRC), Nairobi, Kenya. The results will be disseminated through workshops, policy briefs, peer-reviewed publications, and presented to local and international conferences.

**Trial registration:**

PACTR201811870943127; Pre-results. 26 November 2018.

**Electronic supplementary material:**

The online version of this article (10.1186/s12889-019-7497-3) contains supplementary material, which is available to authorized users.

## Background

Malnourished children, particularly those with severe acute malnutrition, have a higher risk of death from common childhood illness such as diarrhea, pneumonia, and malaria. Nutrition-related factors contribute to about 45% of deaths in children under 5 years of age [1, 2] The direct relationship between illness and malnutrition in children is well documented and evidence has shown that malnutrition is a factor in more than half of the children who die after the first month of life [2, 3]. Pneumonia, diarrhea and malaria are the leading cause of child mortality and malnutrition contributes to more than a third of all child deaths.

In Kenya, the trends of malnutrition among children under the age of 5 years over the last 15 years is suboptimal with slow improvement. According to estimates from the 2014 Kenya Demographic and Health Survey (KDHS), more than a quarter (26%) of children under five were stunted, 11% were underweight and 4% were wasted. In Northern Kenya counties where the prevalence of malnutrition is high, the prevalence of stunting, wasting and underweight has been consistently higher for a long time.

Despite the evidence, in many developing countries, a myriad of health system bottlenecks and demand-side barriers limit the reach of health and nutrition interventions leaving out many children in need [4, 5]. As a strategy to address these challenges, WHO and UNICEF issued a joint statement in 2004, supporting the clinical management of common childhood illnesses, including diarrhea and pneumonia at community levels [3, 6]. WHO also issued program guidelines for the home management of malaria [7, 8]. In 2012, elements of these community-based services were brought together in a package known as integrated community case management (iCCM), World Health Organization (WHO) and UNICEF also issued a joint statement of iCCM support [9]. The iCCM is a strategy to extend case management of childhood illness beyond health facilities so that more children have access to lifesaving treatments. The strategy also aims to improve equity by reaching the hard-to-reach and underserved segments of the population [10].

Cognizant to global initiative, the Kenyan Government, after elaborate consultations with stakeholders and aligning within its community health strategy, has launched a five-year National Framework and Plan of Action for Implementation of iCCM in 2013 [11]. The iCCM strategy encompasses the community treatment of malaria, pneumonia and diarrhea by trained and properly supervised community health volunteers [11, 12]. The strategy also involves some aspects of maternal, child health and nutritional screening for children under 5 years and referral of cases of malnutrition to health facilities for further management.

Research evidence has shown that about 80% of children with severe acute malnutrition (SAM) who have been identified through active case finding, or through sensitizing and mobilizing communities to access decentralized services themselves, can be treated at home [13, 14]. Thus, integration of management of acute malnutrition with iCCM would have the potential to maximize synergy of the interventions and improve efficiency of resource utilization.

However, provision for home or community-based management of acute malnutrition is not included as part of the Kenyan iCCM framework. Furthermore; little is known whether the value obtained from integrating the management of acute malnutrition within iCCM is worth the resources invested in the implementation of this intervention program. Evidence on the potential impact as well as practical experiences on how to integrate nutrition (including community-based management of acute malnutrition) as part of an iCCM package is limited or scantly documented.

Therefore, we aim to test the feasibility and effectiveness of integrating management of acute malnutrition (both MAM and SAM) into iCCM including treatment outcomes, CHVs performance, and coverage elucidating the enabling factors and challenges of integration. Such local evidence could inform policy and strategies for scaling up effective coverage of nutrition interventions, and therefore will have a paramount significance in informing the potential impact, costs and cost-effectiveness of linking nutrition with iCCM in Kenya.

### Research questions

#### Primary research question

The primary research question that the study seeks to answer is: Does integrating management of acute malnutrition (including MAM and SAM) into iCCM improve coverage, quality of care, and treatment outcomes of children 6–59 months with acute malnutrition?

#### Secondary research questions

The study will also explore a number of secondary research questions focusing on the process variables. The secondary research questions include:

1. Can CHVs effectively manage acute malnutrition at the community level?
  To what extent can CHVs screen, give treatment and manage ready-to-use therapeutic food (RUTF), ready-to-use supplementary food (RUSF) and other iCCM commodities at community level?
  What are the experiences of the CHVs in using the simplified CMAM tools?
2. What is the effect of integrating acute malnutrition on the CHV’s performance, in regards to workload and quality of care in the management of primary iCCM illnesses (diarrhea, malaria, and pneumonia)?
3. What are the enabling factors and challenges in integrating management of acute malnutrition into iCCM?
4. Is the integration of management of acute malnutrition into iCCM cost effective compared to the baseline system (traditional facility-based approach)?

### Conceptual framework and operationalization

**Fig. 1:**
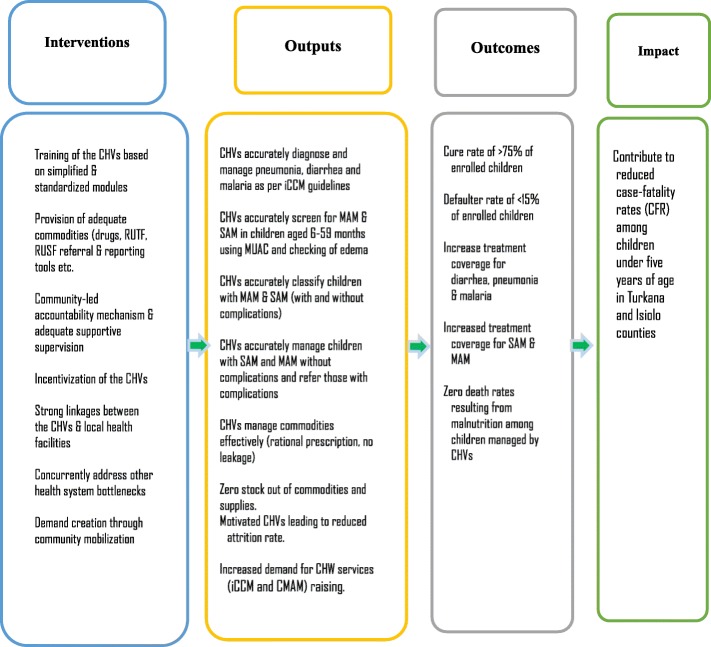
Study’s conceptual framework

This implementation research seeks to test the conceptual theory of change as shown in Fig. 1 Under the iCCM, we argue that an effective training of CHVs to screen, treat malnutrition combined with an adequate provision of commodities: RUTF, RUSF, adequate supportive supervision will trigger a timely case-finding of MAM/SAM cases which will result to an early treatment to uncomplicated cases of malnourished children, hence increased treatment coverage. Furthermore, a full adherence of the CHVs and individuals to the treatment protocol will yield a high recovery rate, little defaulting and reduced case-fatality rate.

## Methods/Design

### Study design

This is a two-arm, parallel groups, Cluster Randomized Controlled Trial (CRCT) implementation study. The intervention arm involves deployment of CHVs trained to screen, diagnose and treat moderate acute malnutrition (MAM) and severe acute malnutrition (SAM) in to ten randomly selected clusters (CUs); while in the control arm, CHVs will provide the standard of care that include screening and referral of MAM/SAM cases to the nearby health facility for treatment by healthcare professional. An independent trial steering committee and a national level technical advisory committee (TAG), will oversee the trial, review progress, and decide on any changes to the protocol. A study statistician will also provide data advice to the trial steering committee, as necessary.

### Study setting

This trial will be conducted in Turkana and Isiolo Counties of Northern Kenya. The sites were identified based on a pre-set criteria including, but not limited to:

- High burden of acute malnutrition in the area. Being arid and semi-arid lands (ASALs), both Turkana and Isiolo Counties have high prevalence of acute malnutrition. This will make it possible to get adequate sample size and also provide opportunity for CHVs to practice and gain experience in case management.
- Long distances to health facilities; thus, justifying community case management of acute malnutrition.
- Existence of community units (CUs) with CHVs that have already been trained on the basics of community health strategy modules.
- A supportive County Government and where the county health management team (CHMT) has clear plans for rolling out the community health strategy. Both Turkana and Isiolo CHMTs have identified iCCM and CMAM as priority interventions in their areas.
- Preferably where there is existing supply chain system for RUTF and RUSF established through a partnership between the County Government and UNICEF, World Food Program (WFP) and Kenya Medical Supplies Authority.
- Presence of Action against Hunger (ACF), Save the Children, or UNICEF within the county to supplement the Ministry of Health (MoH). Apart from reducing the operational costs of the research, the on-the-ground presence will make it possible to rapidly roll out the project (without having to spend time on setting up in a new area). Save the Children is active in Turkana while ACF is implementing nutrition programs in Isiolo County
- Easy to access and relatively secure county to enable consistent implementation and ongoing monitoring and evaluation of the project. Ease of access will be critical as some of the project partners such as MoH’s New-born, Child and Adolescent Health Unit (NCAHU), Nutrition and Dietetics Unit (NDU) and KEMRI will be based in Nairobi but expected to be making regular visits to the study sites. Both Counties are accessible either by road or air.

### Description of the counties selected for the trial

#### Turkana County

Turkana County, situated in North Western Kenya, is the largest of the 47 counties in Kenya with a total area of 77,000 Km^2^ administratively divided into seven sub counties namely; Turkana Central, Loima, Turkana South, Turkana East, Turkana North, Kibish and Turkana West. According to the latest, 2009, Kenya Population and Housing Census (KPHS) [15] and statistical estimates, the county has a population of 855,399, and expected to grow to 1,427,797 by the year 2017. The population of children under 1 year and those below 5 years is projected to reach 25,922 and 186,243 respectively by 2017.

Turkana is drought-prone and experiences frequent, successive and prolonged droughts virtually every year. Turkana’s harsh environment and historic underdevelopment has led to the highest poverty rate in Kenya at 94%, with significantly lower indicators for human development and service coverage than the rest of the country. According to the 2009 KPHS, less than one in five (18%) of the population can read and write, only 9% roads were tarmac and less than one in five households had access to improved sanitation [15]. The average distance to a hospital, health centre or dispensary is 50 km. Serious gaps in human resources further complicate service availability. Doctor to population ratio stands at 1:70,000 while nurse to population ratio is 1:5200 [16].

According to the SMART survey conducted in June 2015 covering all the livelihood zones in Turkana county (pastoral, agro-pastoral and formal employment/business/petty trade), about one in four (25%) children in the county are acutely malnourished. The rates of acute malnutrition in Turkana Central/Loima, North and South/East indicated a *very critical* nutrition situation, while the nutrition situation in Turkana West was classified as *critical*. The weighted Global Acute Malnutrition (GAM) for Turkana County was 21.2% and SAM rate was 5%.

#### Isiolo County

Isiolo County is located in the North Eastern part of Kenya and borders Marsabit County to the North, Samburu and Laikipia Counties to the West, Garissa County to the South East, Wajir County to the North East, Tana River and Kitui Counties to the South, Meru and Tharaka Nithi Counties to the South West. The county covers an area of approximately 25,700 km^2^ and has an estimated population of 143,294 of which children aged 0–59 months are estimated to be 31,163. It consists of three sub-counties namely Isiolo, Garbatulla and Merti.

The county is characterized by recurrent droughts, hot and dry climate with low and erratic rainfall patterns, and is prone to food insecurity predisposing vulnerable groups such as children aged 6–59 months to malnutrition. The county has two rainfall seasons; long (mid-March to May) and short rain (October–December) season. The county just like the other ASALs counties has poor child health indicators. The most recent SMART survey reported an increase in fever from 25% in 2015 to 43% in 2016, acute respiratory infection from 58 to 64% and diarrhoea from 16.3 to 25%. Additionally as expected with the high prevalence of child health diseases, the county has poor nutritional indicators. The SMART survey reported a GAM prevalence of 3.8% [95% CI, 2.3%- 6.4] and SAM prevalence of 0.4% [95% CI, 0.1%-1.6]. Isiolo County has weak community health systems structure and these will need to be strengthened as part of implementation of this protocol**.**

### Sample size estimation

We used recovery rate from acute malnutrition as a primary outcome to estimate the sample size required for the study. So, based on the current estimates in the study area [17], we set the primary outcome (recovery rate) at 75%, and posit that there is truly no difference between the standard (CHVs are not treating MAM/SAM cases) and experimental treatment (CHVs are treating MAM/SAM cases). Hence, sample size estimates is based on the assumption of non-inferiority [18] in CRCT - details on sample size calculation is given in Additional file 1

To estimate the sample size, we assume an intra-cluster correlation (ICC) of *ρ* = 0.005, one-sided test with a confidence interval of 95%, a power of 80%, cluster size of 24 cases of MAM/SAM, and an attrition rate of 5%. We assume a non-inferiority limit of 15%. Thus, a total of 120 under-five children (6–59 months) per arm, with MAM/SAM (240 MAM/SAM cases in total) will be required. So, for a cluster size of 24, we need 10 clusters or Community Units (CUs) to get a total sample of 240 under five cases (120 from intervention and 120 from control arm).

Putting separately, in each county (Turkana and Isiolo), we will recruit 120 children in the control group and 120 children in the intervention group. The cluster size per community units is 24 children. To ensure balance between the control group and intervention group with respect to baseline characteristics, we will use a paired-matching design where each control group will be matched with intervention group which has similar characteristics using the data derived from the baseline activities.

In summary, at the baseline (phase I - before intervention) a new cohort of 240 children with MAM/SAM will be recruited and followed-up in all of the 10 community units. Similarly, another cohort of 240 children will be recruited and assed for study outcomes. The detailed estimates of sample size is found in the Appendix. Table 1 illustrates the design.

**Table 1:** Illustration of the study design

Baseline (phase 1)-cohort (A)		Endline (phase 2)- cohort (B)	
Control (#)	Intervention (#)	Control (#)	Intervention (#)
CUo1 (24)	CU_I_1 (24)	CUo1 (24)	CU_I_1 (24)
CUo2 (24)	CU_I_2 (24)	CUo2 (24)	CU_I_2 (24)
CUo3 (24)	CU_I_3 (24)	CUo3 (24)	CU_I_3 (24)
CUo4 (24)	CU_I_4 (24)	CUo4 (27)	CU_I_4 (24)
CUo5 (24)	CU_I_5 (24)	CUo5 (24)	CU_I_5 (24)
Total = 5 CUo (120 children)	Total = 5 CU_I_ (120 children)	Total = 5 CUo (120 children)	Total = 5 CU_I_ (120 children)

CU stands for community units. CUo, CU_I_ are community units in the control and intervention, respectively

### Sampling procedures

Households with a case of MAM or SAM deemed eligible to the survey will be selected using two-stage cluster sampling technique. In the first stage, we will randomly select CUs based on their level of functionality (existence of at least one CHEW, at least one dialogue day in the last 3 months, at least 10 CHVs in the community unit…etc.) and distance to the nearest health facility; where we shall consider a distance of greater than 5 km to the nearest health facility. At the second stage, a random sampling will be used to select the 24 cases of MAM/SAM in each community unit from sampling frame of MAM/SAM diagnosed by CHVs. For instance with regard to Isiolo Sub-County in Isiolo County, 10 CUs are classified as functional and five kilometers from the nearest health facility meanwhile for Loima Sub-County in Turkana County, we have 23 communities units which are classified as functional. Hence, we will randomly select 10 community units in Loima Sub-County using Stata. For Isiolo County the same approach is used to select the 10 community units.

#### Inclusion/exclusion criteria

To be included in the study, the following criteria highlighted in Table 2 should be met.

**Table 2:** Inclusion and exclusion criteria

CHVs Inclusion/ exclusion Criteria	Under-five Children Inclusion/ Exclusion Criteria
Inclusion criteria • CHVs who have been recruited and trained as CHVs by the MoH community strategy and willing to undertake and devote more of their time to the study	Inclusion criteria • Children 6 to 59 months presenting with SAM (a MUAC of < 115 mm or presence of bilateral pitting edema, without complications) or MAM (MUAC of 115 to 125 mm). • Parents/caretakers willing to participate in the study
Exclusion criteria • CHVs who have discontinued their community services or move out of the study area	Exclusion criteria • Parents/caretakers who are not willing to consent/ participate in the study.

#### Outcome measures

The overarching our outcomes of integration are seamless service provision, maximized synergy and improved efficiency and effectiveness. The following list shows key primary and secondary outcome indicators.

### Primary outcomes

- Cured rate
- Case-fatality rate/Death rate
- Defaulted rate
- Non-cured rate
- Average length of stay
- Average weight gain
- Coverage rate

### Secondary outcomes

- Treatment of fever, pneumonia and diarrhea.
- Quality of care (CHVs performance and workload)
- Challenges and enablers in the effective integration of management of acute malnutrition into iCCM
- Cost-effectiveness analysis
  - Cost per child recovered (from both a program and a societal perspective)
  - Incremental cost-effectiveness ratio for ICCM model
  - Range of costs within the sensitivity analysis

### Intervention protocol

#### Criteria for admission

At admission, the CHVs will only screen under five children (aged 6–59 months) and carryout preliminary examination for any danger signs. This will be followed by further examination for complaints including cough/cold, diarrhea, dehydration and fever, which will be followed by anthropometric measurements to classify the child in relation to nutritional status either MAM, SAM or normal. A special five color-band MUAC tape will be used to allow identification.

Children with MUAC < 9.5 cm and/or less than 4 kg on the dosage scale, with/without bilateral pitting oedema, those who fails the appetite test or show presence of iCCM danger signs or other illnesses other than iCCM diseases (diarrhoea, malaria, pneumonia), sick neonates (less than 1 month) and malnourished children less than 6 months, will be subject for referral to the health facility. However, those sick children below 2 months and caregivers who fail to consent will be excluded at the recruitment and only referred to the health facility.

If MUAC is < 11.5 cm, the child will be classified as SAM; before admitting the child directly for treatment, do the following: children presenting a MUAC of < 11.5 cm will be defined as with SAM while MAM will be defined as MUAC of between 11.5 cm and 12.5 cm. The CHVs will then conduct physical examination of MAM and SAM cases. At this stage, examination for any oedema not observed during assessment of danger signs will be detected. Appetite test will then be conducted setting the stage for either referral or exclusion. Wait for up to 30 min before deciding the child has poor appetite. If the child takes significant quantities as indicated in the Table 3, interpret as the child has good appetite. If no complications and good appetite, the child will be admitted for community-based management of malnutrition.

**Table 3:** Appetite test interpretation

Weight (kg)	Sachet portion the child needs to finish to pass appetite test
4–6.9	1/4 to 1/3
7–9.9	1/3 to ½
10–14.9	1/2 to ¾
15–29	3/4 to 1

For SAM children, the CHVs will give first dose amoxicillin dispersible tablets (DT) on the spot and twice for 7 days before the next visit. If rapid diagnosis test (RDT) is positive for malaria, CHVs will give one dose on the spot and twice daily for 3 days (the prescriptions will clearly be explained to the mother). Then RUTF will be given to the mother/caregiver and explain the daily intakes throughout until the next visit as explained. Table 4 provides details on RUTF that should be given to children based on their weight.

**Table 4:** Dosing chart for RUTF

Child’s weight (kg)	Packets per day	Packets per week
4.0–4.9	2	14
5.0–6.9	2.5	18
7.0–8.4	3	21
8.5–9.4	3.5	25
9.5–10.4	4	28
10.5–11.9	4.5	32
≥ 12	5	35

With regards to MAM cases, albendazole and folic acid (for weight) will be given on the spot and further dosage explained to the mother/caregiver. Then CHVs will give RUSF for the children for 7 days until the next visit. Mother/caregivers will be advised to respect follow-up, breastfeeding and diversification of feeding in addition to RUSF. The CHVs will use the following dosing chart to administer.

#### Follow-up stage

At follow-up, CHVs will ask and seek for general danger signs and examine any complaints on the child. In addition, the systematic evaluation will be conducted to examine any cough, fever or diarrhea following the standard treatment and follow-up protocol. Anthropometric measurement weight and MUAC will be taken to determine any changes (gains) in the child. The CHVs will further conduct systematic evaluation to look for edema not detected at the general danger signs and appetite test. Children with either danger signs, failed appetite test, weight loss, diarrhea will be referred to the health facility, otherwise the CHVs will conduct treatment for MAMA or SAM as appropriate.

For SAM cases, during follow-up the RUTF will be provided for the next 7 days and if RDT is positive, ACT will be provided on the spot and twice daily for 3 days. Adelbanzole will be given if during the second visit the child is aged more than 12 months. In the case of MAM, the CHVs will give folic acid and continue RUSF for the next 7 days. The diversification and breastfeeding should be emphasized again at this point.

Furthermore, during follow-up all SAM children will be subject to weekly visit to CHVs for MUAC measurement and weight using the dosage scale, and collection of RUTF as per dosage scale will continue for a maximum of eight weekly visits if the child is progressing well, otherwise referral will be recommended in case of non-response to treatment. On the other hand, MAM children will be subject to bi-weekly (every 2 weeks) visits for MUAC measurement and collection of RUSF (1 sachet per day for 16 weeks for a maximum of eight visits if child is progressing well, otherwise the child needs to be referred). Table 5 outlines the follow-up procedure for both MAM and SAM.

**Table 5:** Follow-up procedure for both MAM and SAM

Follow up	Discharge criteria		Action
1) SAM, MUAC < 11.5 cm			
Weekly visit to CHV for MUAC measurement and weight using the dosage scale and collection of RUTF as per dosage scale Maximum of 8 weekly visits if child is progressing well Two consecutive MUAC and/or weight measurements with no change or deterioration	cured	MUAC≥12.5 cm 2 consecutive green on MUAC	Discharge as cured
	defaulter	Misses 3 consecutive visits	Exclude from the study, Trace and admit as return defaulters but not for study
	Treatment failure	Two consecutive MUAC and/or weight measurements with no change or deterioration	Refer to health facility for further investigation
	/Non-response	Two consecutive MUAC measurements with no change of deterioration	
	Lost to follow up	Misses 3 consecutive visits and cannot be traced	Exclude from the study
2) MAM; 11.5 cm ≤ MUAC < 12.5 cm			
Bi-weekly visits (every 2 weeks) for MUAC measurement and collection RUSF (1 sachet per day for 16 weeks Maximum of 8 visits if cyhild is progressing well	cured	MUAC≥12.5 cm 2 consecutive green on MUAC measurement	Discharge as cured
	defaulter	Misses 3 consecutive visits	Exclude from the study, trace and admit as return defaulters but not for study
	Treatment failure/non-response	Two consecutive MUAC measurements with no change; deterioration on MUAC	Refer to health facility for further investigation
	Lost to follow up	Misses three consecutive visits and cannot be traced	Exclude from the study

All the activities during this intervention will be clearly documented using primary data collection tools and will be collated and for further analysis on the effectiveness of ICCM-CMAM integration.

### Data collection procedures

#### Quantitative data

Quantitative data will be conducted at baseline to establish coverage of SAM and MAM treatment as well as other relevant nutrition and iCCM indicators. Data on treatment outcomes will be collected during the implementation. Further, end line quantitative data collection will be done to assess the coverage of SAM and MAM treatments as well as other relevant nutrition and iCCM indicators.

These data will be collected via quantitative household surveys. Building on our extensive experience with household nutrition surveys and iCCM, We shall use questionnaires to collect detailed information on socio-demographic and economic characteristics of the household, child characteristics including health, nutrition status and standard anthropometrics in every time period. Anthropometric data, will include child’s weight, presence of oedema, height and mid-upper arm circumference will also be collected, using standard protocol [19]. Additionally, CHVs will also be interviewed to obtain data that shall inform of their understanding and practice of the laid out protocol. The same information will be compared with data collected from mothers or caregivers of children in the clusters to gauge the treatment regime received compared to the laid out practice. Altogether an understanding of the effectiveness of this service will be obtained. These questionnaires to be used will be pre-tested before fielding the final survey. Furthermore, in the Kenya context, at the level of health facility, there are registers which are used to follow-up children with MAM/SAM cases. Those registers provide valuable information on the outcomes (recovery, defaulter and death). But, for the community-based management of malnutrition, we will use similar registers to record all the management of MAM/SAM cases which will also provide information on our outcomes. We will setup a tracking system which help us to randomly select our sample size of MAM/SAM cases and follow-up them till 4 months period at each time point.

#### Costing data

Collection on costing data for integrating nutrition into iCCM will be carried out from the health system’s perspective and focus on the costs incurred by the system (e.g. government, donors) for the delivery of this health intervention. Furthermore, we will explore the societal perspective by including costs for the caregiver e.g. the caregivers’ travel costs and lost labor time, societal/opportunity costs. Other research costs such as salaries of personnel doing research (field monitors/supervisors) will not be taken into account.

In this health intervention program, the costs incurred could be summarized into three components.

*Start-up costs or pre-implementation costs*. These are costs which are borne by the provider of the iCCM intervention program to launch the intervention. These include: community outreach (mass media campaigns, social mobilization, focus group discussion, behavioral change communication etc.), training of CHVs who will implement the intervention, venue rental costs, material/office supplies used for the training, lodging costs of trainer and transport costs (fuel, vehicles), refreshments, allowances/per diems, CHVs start-up medicines and tools kit. The financial report will be used to capture these costs.

*Recurrent costs* are costs of resources incurred repeatedly and consumed within a year of procurement. These include: human resources (salaries, stipends of CHVs, supervision of the CHVs), consumables (pens, office supplier, reporting tools etc.) and operating costs of vehicles (fuel, utilities). We will also use time and motion survey to estimate the weekly workload of CHVs and the average time spent in implementing the intervention. Furthermore, we will also collect information on the cost of drugs used for the diagnosed, treatment of diarrhea, pneumonia, malaria and diseases of malnutrition. The source of these activities is the financial report, time and motion survey, and direct interview with the provider of the intervention.

*Capital costs* are costs borne for inputs which have a useful of life longer than a year such as vehicles, buildings and equipment. The resources identified as capital costs are calculated taking into account that these resources are used for several years and their alternative uses (opportunity cost). We therefore estimate the capital costs on an annualized basis using the current value of these resources, useful life, discount rate of 3% and animalization factor.

The program manager or institutional perspective is the three components listed above. The societal perspective is more global since it includes the three components of cost listed above and other household costs incurred for participating in community-based management of malnutrition (time taken to feed the child suffering from MAM/SAM, additional food purchased to feed the child and other related cost incurred).

In order to account for the inflation, all the costs will be converted in constant prices using the GDP deflator for year 2019 for Kenya. Our base year of costing exercise will be 2019. Furthermore, we will explore whether integrating management of acute malnutrition into iCCM is cost effective using the decision rule based on WHO-CHOICE (Choosing Interventions that are Cost-Effective).

#### Qualitative data

We will conduct qualitative data collection in three stages: pre-intervention, midline and post intervention qualitative data collection. For each of the stages, we will conduct focus group discussions (FGDs), in-depth interviews (IDIs) and key informant interviews (KIIs) as well as observation of care provision to assure quality of services. The number of interviews per stage and sub-group to be interviewed as well as the sample size will mainly depend on the level needed to achieve saturation of concepts. However, we anticipate the following number of interviews per stage: 8 IDIs, 8 FGDs, and 14 KIIs per county. We expect that each FGDs will have 8 people. Table 6 gives an overview of the sample size for qualitative data collection.

**Table 6:** Summary of proposed KII, IDI and FGDs, in each county (Turkana and osiolo)

Method	Participants	Interviews/discussion sessions	Participants (#)	Total discussion sessions (#)
IDIs	Mothers			
	• In program, • Not in the program • Defaulters	8	1	8
		8	1	8
		8	1	8
FGDs	Mothers	1	6	6
	Men (fathers)	1	6	6
	Grandmothers	1	6	6
	CHW	1	6	6
KII	Community leaders & others	6	1	6
	Health professionals • Doctors, Nurses & nutritionist	2	1	2
	County and national level CHMT and Policy/decision makers	2	1	2
	Implementing partners (ACF/Save the children)	2	1	2
Total				60

For the qualitative data collection, purposive sampling will be used to sample women of reproductive age with children suffering from acute malnutrition, fathers of children being treated for acute malnutrition, other community members including grandmothers, health care providers, community leaders and policy/decision makers in the Ministry of Health, County health management teams, county executive officer, county director of health, implementing partners and other key organizations in the study areas.

### Pre-implementation qualitative data collection stage (baseline)

Rapid qualitative methods including in-depth interviews, FGDs and KII will be conducted with various stakeholders. Targeted women will be those whose children have been /are being treated for acute malnutrition or are suffering from acute malnutrition, their spouses and other men whose children have been treated/are being treated for malnutrition, other stakeholders including implementing partners, Ministry of Health officials, County government and local community leaders.

The qualitative study will seek to answer questions regarding general knowledge attitude and practices with regards to child feeding and current treatment of MAM/SAM, challenges that hinder treatment of acute malnutrition, cost and time implications of the new model of treatment, and the barriers and facilitators that affect the MAM/SAM treatment outcome measures including average length of stay; average rate of weight gain; cure rate; mortality rates; default rates and transfer rate. The study will also qualitatively seek to establish the views of CHVs and other health workers on the effect of the integration of iCCM and management of acute malnutrition (SAM and MAM) on their workload as well as its likely effect on the quality of standard iCCM care. For the implementing partners, policy and decision makers, we will focus mainly on their recommendations for adaptation of the integrated program and their perceived role in it.

#### Implementation phase qualitative data collection

During the implementation phase, qualitative data will be collected at mid-term whereby we will conduct in-depth interviews, key informant interviews and focus group discussions with women/ caregivers in the program, on their experiences with the integrated package and their satisfaction as well as suggested potential areas of improvement. We will also conduct focus group discussions with CHWs and key informant interviews with other stakeholders (implementing partners and the Ministry of Health Officials including the CHMT and Health Facility Workers) on their views on the progress of the integrated program including facilitating and limiting factors, and the cost and time implications of the intervention. The information gathered will be useful in engaging the stakeholders including the implementing partners, the Ministry of Health, UNICEF and WFP during the remaining section of the implementation phase in order to minimize potential risks and counter challenges experienced and to maximize on the potential benefits.

#### Post-implementation qualitative data collection

We will conduct qualitative interviews among women whose children were treated for acute malnutrition during the implementation period, and men (fathers of the children) as well as other community members to assess their experience and satisfaction with the integrated program within the period of the implementation, and potential areas of improvement, observed advantages and challenges and perceived effects of the integrated program including adherence to the treatment given, recovery rate and their relationship with the CHVs during the implementation phase. We will also conduct interviews with the CHVs to determine their experiences in implementing the integrated package including challenges and facilitators; their views on its effect on their workload; effect on the quality of care given; their views on their capability to administer treatment; and their suggestions on the possible areas of improvement.

### Training of research assistants and piloting

Data will be collected by qualified and well trained data collectors in line with APHRC’s training requirement. Qualitative data collection will be undertaken by trained field interviewers with college or higher level of education (preferably in nutrition or health related field) and with previous experience in qualitative data collection in arid and semi-arid lands. Quantitative data will be collected by carefully trained field workers with a college certificate and previous experience in quantitative and qualitative data collection. All field workers will be expected to be fluent in both English and Swahili and the local language with a preference given to interviewers residing in or near the study area. After recruitment, research experts on the different methods of research as detailed above from within the team will conduct a week-long training of field interviewers on ethics for research with human participants, data collection procedures, ensuring good quality data, and reporting. Consistent check-ins with the interviewers will be used as an approach to ensure high quality data is captured.

### Management of data quality during field work

To ensure that high quality quantitative data is collected, validation rules, constraints or checks (skips) in the questionnaire will be embedded within the data collection electronic software during programming of the tool. This will allow the interviewers to quickly notice missing data and implausible or out of range values. The data collection program will also be tested severally prior to data collection. Regular spot checks by the project team with field interviewers, and an automated routine check on data completeness and discrepancies will also be implemented to enhance data quality. Interviewers will be responsible for following-up on any inconsistencies or errors in the data with their participants as/when needed.

To ensure that high quality qualitative interviews are conducted, the research team will sit-in on a select number of interviews, also regularly review the data collected for quality. Regular debriefing sessions with the interviewers will also ensure that emerging ideas are followed-up on in subsequent interviews. In addition, transcripts will undergo data verification by checking them to confirm accuracy against the original audio-recordings.

### Data processing and analysis

#### Quantitative data management, storage and analysis

In a non-inferiority in CRCT, we will first analyze the findings using non-parametric namely the adjusted Chi-squared test for clustered binary such as recovery rate, case fatality and defaulter rate. For continuous outcomes, we will use t-test for clustered data namely for weight gain. Furthermore, for our primary outcome we will use an econometric model namely a Probit model where the dependent variable is the recovery rate and covariates are the treatment variable (one for the intervention and zero for the control), age of the children, gender of the children, vaccination status (unvaccinated, partially vaccinated, fully vaccinated), overall length of stay, breastfeeding, co-morbidities at admission (malaria, anemia, pneumonia, measles and giardiasis), admission status (new admission, readmission, admission after default), education of the mothers, income of the mothers etc.

All digital project data will be stored in encrypted computers or laptops that are password protected, with regular back-up procedures to the server put in place to ensure that the data is not lost. All survey data will be collected via password protected tablets and uploaded to a secure cloud-based data depository after completion of the survey. Data will then be deleted from the tablets. Non-anonymized data will be stored on a secure, password projected server made accessible only to the core research team. All data will be anonymized before being made available to other project staff, ensuring the confidentiality of data following collection.

Quantitative data will be analyzed using STATA, SPSS and/or MLWin files. Prior to data sharing, it will be anonymized. The primary aim of the analyses conducted will be to answer the research questions outlined in the protocol. Quantitative data analysis will mainly be descriptive. Qualitative interviews will be audio-recorded on encrypted recorders, transcribed verbatim (and translated/transcribed as needed), anonymized and stored in digital format (MS Word compatible).

#### Qualitative data analysis

Tape-recorded qualitative data will be transcribed verbatim. Rapid analysis of the transcribed qualitative data collected during the preceding phases for the purpose of informing the succeeding phases will be done through systematic reading through the transcripts.

For further understanding and use of the data including dissemination and scientific writing, data will be coded in NVIVO (QSR International Pty Ltd), to identify primary and meta-codes and major themes. Themes will be identified, with attention to contradiction and diversity of experiences, perception and attitudes across the different interviewees. Analysis across all transcripts will be done thematically [20].

A qualitative software package (NVIVO) will be used to code the qualitative data and support analyses. Thematic analysis will be used to identify specific themes from the individual interviews using NVivo. Coding and interpretation will be undertaken by at least two members of the research team to ensure objectivity and consistency during coding. All transcripts will be anonymized to conceal the identity of the research participants. The anonymized transcripts will be stored in password protected computers, accessible only by the research team.

### Plans for communicating study findings

Engagement, communication and dissemination with academics and wider communities (e.g. key informants, policy makers and media) will take place through knowledge translation and research uptake activities; open access publications; conference presentations; and via a project website and social media platforms. Policy briefs and press releases, workshop with key stakeholders, and academic papers, conference presentations, and research seminars will all be used to widely disseminate study findings.

### Study risks and limitations

Overall, this project is expected to pose no major risks to the participants, in terms of requiring their time and infringement of privacy as the recruitment and cohort follow-up will happen in their homes. We believe that the minimal chance of risk is acceptable in light of the benefits of the research project. Participant informed consent and confidentiality will be adhered to strictly.

Turkana and Isiolo have are considered as hardship areas especially due to infrastructural challenges, extreme weather conditions and language /communication barriers, given the low literacy levels recorded in the two regions. These could be potential challenges in the study implementation. To mitigate these challenges, recruitment of the field team and supervisors will prioritize local community members, to minimize the need for frequent travel (for the infrastructural challenges) and communication challenge due to language barrier. The study team will also consult with other stakeholders working in the two counties and learn from their experiences in managing such challenges.

## Discussion

According to the latest series of evidence from Lancet Maternal and Child Nutrition, good nutrition is a fundamental driver of a wide range of development goals. Nonetheless; in many developing countries, a myriad of health system bottlenecks and demand-side barriers limit the reach of health and nutrition interventions leaving out many children in need. In such communities, affected by repeated drought and famine, availing ready-to-use therapeutic food (RUTF) to children with SAM through a network of community health volunteers (CHVs) or community-level health facilities has been a key strategy to address the challenge.

In Kenya, guided by the National Guideline for Integrated Management of Acute Malnutrition (IMAM) [21], management of SAM and MAM mainly happens at health facility level. However; amid the investments by the government and other non-governmental organization in the implementation of the IMAM program, studies have demonstrated low access and coverage in some parts of the country, particularly arid parts of Northern Kenya has been evident since the last decade.

Our proposed intervention is based on an innovative approach of integrating and simplifying SAM and MAM management through CHWs bring the services closer to the community. Evidences from operational experiences of other countries that effectively implemented iCCM programs shows that solid support in the form of training, supervision, supply and logistics, and functional referral systems are critical to the success of the program. The major challenges that CHV face in implementing iCCM programs include, but not limited to high attrition rates, huge work load, shortage or stock out of commodities, and substandard care quality. These limitations are mainly related to problems with poor planning, fragmented and condition-specific trainings, poor linkage to the health system, inefficient coordination and other constraints of the the health system [22].

To the best of our knowledge, the proposed study is expected to contribute to the limited evidence on effectiveness and cost-effectiveness as well as acceptability of integrating SAM and MAM treatment with the exiting iCCM approaches. The study will also expected to contribute to strengthen the evidence base of developing basic public health services in Kenya and elsewhere in Africa with similar contexts. In addition, this intervention is also expected to have good replicability which can easily be scaled and implemented by similar low profile CHWs with relatively low cost. However; as ensuring the fidelity of such complex interventions is challenging, we have developed a stringent monitoring and reporting system which will be lead and overseen by the TAG. Sustaining the doubts about the effects of integrating SAM and MAM treatment with the exiting ICCM protocol may also be mitigated with the current approach.

### Additional file

Additional file 1: Sample size calculation for non-inferiority cluster randomized controlled trials. (DOC 87 kb)

## Data Availability

Not applicable

## APPENDIX

### Sample size calculation for non-inferiority cluster randomized controlled trials

We follow Rutterford et al. [35]‘s study for sample size calculation of non-inferiority in CRCT. In the current study, we assume that the integration of CMAM into iCCM will yield a recovery rate of 75% and there is truly no difference between the standard (CHV *are not* treating MAM/SAM cases) and experimental treatment (CHVs *are treating* MAM/SAM cases) in terms pf recovery rate. We assume an intracluster correlation (ICC) of *ρ* = 0.005, one-sided 95% confidence interval, a power of 80%, cluster size of 24 cases of MAM/SAM, and an attrition rate of 5%. Lastly, we assume a non-inferiority limit (*d*) **to be 15%.**

Hence, the following formula of the sample size with binary data is:

1 $$ N=\frac{{\left({Z}_{1-\frac{\alpha }{2}}+{Z}_{1-\beta}\right)}^2\left\lfloor {\pi}_s\left(1-{\pi}_s\right)+{\pi}_e\left(1-{\pi}_e\right)\right\rfloor }{{\left({\pi}_s-{\pi}_e-d\right)}^2}\left\lfloor 1+\left(m-1\right)\rho \right\rfloor $$

with, ${Z}_{1-\frac{\alpha }{2}}$ and *Z*_1 − *β*_ the values from the normal distribution holding $1-\frac{\alpha }{2}$, 1 − *β* below it, respectively. *α* and *β* are levels of significance and power, respectively. Where *π*_*s*_ and *π*_*e*_ are the recovery rate in the standard and experimental treatment group, respectively. *ρ*, *m* and *d* are the ICC, cluster size and non-inferiority limit**,** respectively.

If we further posit that *π*_*s*_ = *π*_*e*_ = 0.75, Eq. (1) becomes:

2 $$ N=\frac{{\left({Z}_{1-\frac{\alpha }{2}}+{Z}_{1-\beta}\right)}^22{\pi}_s\left(1-{\pi}_s\right)}{(d)^2}\left\lfloor 1+\left(m-1\right)\times \rho \right\rfloor $$

Using Eq. (2), the total sample size is:

$$ N=\frac{{\left(1.64+0.84\right)}^2}{(0.15)^2}2\times 0.75\left(1-0.75\right)\left\lfloor 1+\left(24-1\right)\times 0.005\right\rfloor $$

$$ N\approx 114.29 $$

If we assume an attrition rate of 5%, then:

$$ {N}_{CRCT}=\frac{114.29\ }{1-0.05}\approx 120 $$

The total number of cluster per arm is:

$$ {N}_{CRCT}=\frac{120\ }{24}=5 $$

We will therefore recruit 120 MAM/SAM cases for each group. The cluster size of children in each of the five equally sized clusters per arm is 24 children per clusters. This process will be done at the baseline (240) and endline (240).

## Abbreviations

AC: Action Against Hunger
ACT: Artemisinin Combination Therapy
APHRC: African Population And Health Research Center
CHEWs: Community Health Extension Workers
CHMT: County Health Management Team
CHVs: Community Health Volunteers
CUs: Community Units
DT: Dispersible Tablets
FGDs: Focus Group Discussions
GAM: Global Acute Malnutrition
iCCM: Integrated Community Case Management
IDIs: In-Depth Interviews
IMAM: Integrated Management of Acute Malnutrition
KIIs: Key Informant Interviews
MAM: Moderate Acute Malnutrition
MNCHN: Maternal, Newborn And Child Health And Nutrition
MoH: Ministry of Health
MUAC: Mid-Upper Arm Circumference
NCAHU: Newborn, Child And Adolescent Health Unit
NDU: Nutrition and Dietetics Unit
ORS: Oral Rehydration Solution
OTP: Out-Patient Therapeutic Program
RDTs: Rapid Diagnostic Tests
RUSF: Ready to Use Supplementary Food
RUTF: Ready to Use Therapeutic Food
SAM: Severe Acute Malnutrition
SFP: Supplementary Feeding Program
SMART: Standardized Monitoring and Assessment of Relief and Transitions
SQUEAC: Semi-Quantitative Evaluation of Access And Coverage
TAG: Technical Advisory Group
UNICEF: United Nations Children’s Fund
WFP: World Food Program
WHO: World Health Organization
