# Supplementary material for: Integrated and simplified approaches to community management of acute malnutrition in rural Kenya: a cluster randomized trial protocol
Source: BMC Public Health. 2019 Sep 11;19:1253. doi: 10.1186/s12889-019-7497-3 (PMC6739921; doi:10.1186/s12889-019-7497-3)
Supplement: Supplementary file 1 — Sample size calculation for non-inferiority cluster randomized controlled trials. (DOC 87 kb) [file 12889_2019_7497_MOESM1_ESM.doc]

| **ENGLISH CONSENT** |
| --- |
| **AFRICAN POPULATION AND HEALTH RESEARCH CENTER**  **PROJECT TITLE:** Implementation Research on Linking Management of Acute Malnutrition and iCCM  **Participant Information and Informed Consent Form for CHVs**  **Participating in the quantitative interview.** |

**Introduction**

Hello. My name is……………..(*Your name*)………………………. and I am part of a research team from the African Population and Health Research Center (APHRC) led by Dr Kimani . We will be conducting a study titled ‘*Implementation Research on Linking Management of Acute Malnutrition and iCCM’,* employing an approach with a strong community component in order to reach sick and malnourished children who face barriers to accessing treatment. Integrated Community Case Management (iCCM) is a strategy that utilizes community health volunteers (CHVs) to diagnose and treat multiple conditions, most commonly pneumonia, diarrhoea and malaria, in children under-five years. The findings of this study will then be shared with key decision and policy-makers to help them make the best decisions on interventions that could make treatment and management of malnutrition in this community and in the entire country easier and effective.

**Explanation of Procedures**

As part of this study you will participate in a one-on-one discussion that will take about 60 minutes. First, we will ask you some questions about yourself and where you live, and your work. We will seek to understand from your general knowledge attitude and practices with regards to child feeding and current treatment of malnutrition, challenges that hinder treatment of acute malnutrition, and the barriers and facilitators that affect the treatment and management of malnutrition. We shall also seek to understand your work with regards to the treatment and management of Malnutrition and other child hood diseases in this community.

After this interview, our field interviewer will follow-up with you on regular basis, to observe your home visits and collect data related to treatment and management of malnutrition in children from the households assigned to you. We will request that you freely talk about anything you think is important for us to know related to this study.

**Voluntary participation**

Your participation in this interview is voluntary, and if you choose not to participate, you will NOT be treated with prejudice. If at any point in time you feel uncomfortable about any of the questions, you do not have to answer them. If you feel you do not want to continue participating, you can also withdraw without any consequences or penalty and you will not be asked to explain your reasons for withdrawing.

**Confidentiality**

The information that you provide during the interview will be kept private. The only people who will have access to the information you provide are members of the research team. Your name will not be used in documents, reports, or publications related to this research. The data from the interview will be labelled with code numbers. All study documents will be kept in locked file cabinet in the principal investigator’s office at APHRC. Digital information will be stored on encrypted/password protected laptops or tablets and on a protected server at APHRC in a manner that is not traceable to you. You will not be named in any reports and no one other than authorized study personnel will be able to access your information. We highly value your participation in the study and will avoid compromising your anonymity. We intend to delete any electronic data 10 years after data collection.

**Benefits, Harms, and Risks**

Taking part in the interview may not benefit you directly. However, your participation in this study will inform interventions to treat malnutrition in this community and the country as a whole. There is no anticipated risk or harm that you or your family members will suffer as a result of your participation in this study. You are free to decline to participate or answer any question(s) that you are not comfortable answering.

| **Compensation** |
| --- |

No compensation is provided for participation in this survey interview. We appreciate your time to provide us with information that will be useful in developing interventions that will help in treating/management of malnutrition in children this community and Kenya at large.

**Questions and Your Rights as a Participant**

*Dr Elizabeth Kimani*

*Research Scientist*

*African Population and Health Research Center*

*APHRC Campus, Manga Close, Off Kirawa Road, Nairobi, Kenya*

*P. O. Box 10787- GPO - 00100*

*Tel: 020 400 1000*

This study has been approved by a nationally recognized Ethics Review Committee. If you have questions about your rights as a research participant, or concerns or complaints about the research, you may contact the Scientific Steering Committee Members:

*Ethics & Scientific Review Committee - AMREF Kenya*

*Wilson Airport, Lang’ata Road,*

*Office Tel: 020 6994000*

*Fax: 020 606340*

*P.O Box 30125 00100, Nairobi, Kenya*

***Do you have any questions now?***

**Part II: Certificate of Consent**

| **PARTICIPATING IN THIS STUDY** | **YES**  **(tick)** | **NO**  **(tick)** |
| --- | --- | --- |
| This research study has been explained to me, including risks and benefits, and other important things about the study. I have been given the opportunity to ask questions about the project and I confirm that I understand it. |  |  |
| I understand that all procedures for this study have been approved by the Ethics and Scientific Review Committee of AMREF. |  |  |
| I understand that I will not benefit directly from the research done using the data provided. |  |  |
| I agree to take part in the project as a volunteer. This will include being interviewed and audio recorded. I understand that I may be recontacted about other steps of the research, but that I may withdraw from the study at any time. |  |  |
| **USE OF THE INFORMATION PROVIDED** |  |  |
| I am in agreement that data generated may be made available as stated above and I understand that my responses will be kept strictly confidential and my name will not be linked with the research materials |  |  |
| I am in agreement that the information supplied in the list of questions and the information from the measurements taken may be used as stated above. |  |  |
| I agree that some or all the data may be stored in a data repository and that these may be shared with other researchers according to the processes and procedures of this study by using a study code that de-identifies the data (or preserves the confidentiality of the information provided). |  |  |
| I agree that some or all the data may be stored in a database and that these may be shared with other researchers according to the processes and procedures of this study by using my study code or another code that de-identifies my data (or preserves the confidentiality of the information I provided). |  |  |
| I understand that every time a new study is done using the data, permission will be obtained from the ethics committee for the study to make sure that it is used only for the purposes stated above. |  |  |

|  |  |  |  |  |  |  |  |  |  |  |  |  |  |  |  |  |  |  |  |  |  |  |  |  |  |  |  |  |  |  |  |  |  |  |  |  |
| --- | --- | --- | --- | --- | --- | --- | --- | --- | --- | --- | --- | --- | --- | --- | --- | --- | --- | --- | --- | --- | --- | --- | --- | --- | --- | --- | --- | --- | --- | --- | --- | --- | --- | --- | --- | --- |
| Participant | | | | |  |  |  |  |  |  |  |  |  |  |  |  |  |  |  |  |  |  |  |  |  |  |  |  |  |  |  |  |  |  |  |  |
|  |  |  |  |  |  |  |  |  |  |  |  |  |  |  |  |  |  | Printed Name | | | | |  |  |  |  |  |  |  |  |  |  |  |  |  |  |
|  |  |  |  |  |  |  |  |  |  |  |  |  |  |  |  |  |  |  |  |  |  |  |  |  |  |  |  |  |  |  |  |  |  |  |  |  |
|  |  |  |  |  |  |  |  |  |  |  |  |  |  |  |  |  |  |  |  | / |  |  | / |  |  |  |  |  |  |  | / |  |  |  |  |  |
|  |  |  | Signature/mark/thumb print | | | | | | | | |  |  |  |  |  |  |  |  | Date (dd/mm/yyyy) | | | | | | |  |  |  |  | | | | |  |  |

**If volunteers cannot read the form themselves, a witness must sign here:**

I was present while the benefits, risks and procedures were read to the participant. All questions were answered and the participant agreed to take part in the research.

_______________________ _________________________________________________

Date signed (dd/mm/yyyy) Name and signature of witness

***Declaration by researcher:*** *I have given a verbal explanation of the research project, its procedures and risks and I believe that the participant has understood that explanation.*

|  | | | | |  |  |  |  |  |  |  |  |  |  |  |  |  |  |  |  |  |  |  |  |  |  |  |  |  |
| --- | --- | --- | --- | --- | --- | --- | --- | --- | --- | --- | --- | --- | --- | --- | --- | --- | --- | --- | --- | --- | --- | --- | --- | --- | --- | --- | --- | --- | --- |
| Name of researcher | | | | |  |  |  |  |  |  |  |  |  |  |  |  |  |  |  |  |  |  |  |  |  |  |  |  |  |
|  |  |  |  |  |  |  |  |  |  |  |  |  |  |  |  |  |  |  | | | | |  |  |  |  |  |  |  |
|  |  |  |  |  |  |  |  |  |  |  |  |  |  |  |  |  |  |  |  |  |  |  |  |  |  |  |  |  |  |
|  |  |  |  |  |  |  |  |  |  |  |  |  |  |  |  |  |  |  |  | / |  |  | / |  |  |  |  |  |  |
|  |  |  | Signature | | | | | | | | |  |  |  |  |  |  |  |  | Date (dd/mm/yyyy) | | | | | | |  |  |  |
